# Personality, Attentional Biases towards Emotional Faces and Symptoms of Mental Disorders in an Adolescent Sample

**DOI:** 10.1371/journal.pone.0128271

**Published:** 2015-06-05

**Authors:** Maeve O’Leary-Barrett, Robert O. Pihl, Eric Artiges, Tobias Banaschewski, Arun L. W. Bokde, Christian Büchel, Herta Flor, Vincent Frouin, Hugh Garavan, Andreas Heinz, Bernd Ittermann, Karl Mann, Marie-Laure Paillère-Martinot, Frauke Nees, Tomas Paus, Zdenka Pausova, Luise Poustka, Marcella Rietschel, Trevor W. Robbins, Michael N. Smolka, Andreas Ströhle, Gunter Schumann, Patricia J. Conrod, IMAGEN Consortium

**Affiliations:** 1 Department of Psychology McGill University, Montreal, Quebec, Canada; 2 Institut National de la Santé et de la Recherche Médicale, INSERM CEA Unit 1000 “Imaging & Psychiatry”, University Paris Sud, Orsay, Paris, France; 3 AP-HP Department of Adolescent Psychopathology and Medicine, Maison de Solenn, University Paris Descartes, Paris, France; 4 Department of Child and Adolescent Psychiatry and Psychiatry, Central Institute of Mental Health, Medical Faculty Mannheim, Heidelberg University, Mannheim, Germany; 5 Trinity College Institute of Neuroscience and Discipline of Psychiatry, School of Medicine, Trinity College Dublin, Dublin, Ireland; 6 Universitaetsklinikum Hamburg Eppendorf, Hamburg, Germany; 7 Central Institute of Mental Health, Mannheim, Germany; 8 Medical Faculty Mannheim, University of Heidelberg, Heidelberg, Germany; 9 Neurospin, Commissariat à l'Energie Atomique et aux Energies Alternatives, Paris, France; 10 Institute of Neuroscience, Trinity College Dublin, Dublin, Ireland; 11 Departments of Psychiatry and Psychology, University of Vermont, Burlington, Vermont, United States of America; 12 Department of Psychiatry and Psychotherapy, Campus Charité Mitte, Charité –Universitätsmedizin Berlin, Berlin, Germany; 13 Physikalisch-Technische Bundesanstalt (PTB), Braunschweig und Berlin, Berlin, Germany; 14 Rotman Research Institute, University of Toronto, Toronto, Ontario, Canada; 15 School of Psychology, University of Nottingham, Nottingham, United Kingdom; 16 Montreal Neurological Institute, McGill University, Montreal, Quebec, Canada; 17 The Hospital for Sick Children, University of Toronto, Toronto, Ontario, Canada; 18 Behavioural and Clinical Neurosciences Institute, Department of Experimental Psychology, University of Cambridge, Cambridge, United Kingdom; 19 Department of Psychiatry and Psychotherapy, and Neuroimaging Center, Technische Universität Dresden, Dresden, Germany; 20 Department of Psychiatry and Psychotherapy, Campus Charité Mitte, Charité –Universitätsmedizin Berlin, Berlin, Germany; 21 MRC Social, Genetic and Developmental Psychiatry (SGDP) Centre, Institute of Psychiatry, King’s College London, London, United Kingdom; 22 Department of Psychological Medicine and Psychiatry, Institute of Psychiatry, King’s College London, United Kingdom; 23 Department of Psychiatry, Université de Montreal, CHU Ste Justine Hospital, Montreal, Quebec, Canada; National Center of Neurology and Psychiatry, JAPAN

## Abstract

**Objective:**

To investigate the role of personality factors and attentional biases towards emotional faces, in establishing concurrent and prospective risk for mental disorder diagnosis in adolescence.

**Method:**

Data were obtained as part of the IMAGEN study, conducted across 8 European sites, with a community sample of 2257 adolescents. At 14 years, participants completed an emotional variant of the dot-probe task, as well two personality measures, namely the Substance Use Risk Profile Scale and the revised NEO Personality Inventory. At 14 and 16 years, participants and their parents were interviewed to determine symptoms of mental disorders.

**Results:**

Personality traits were general and specific risk indicators for mental disorders at 14 years. Increased specificity was obtained when investigating the likelihood of mental disorders over a 2-year period, with the Substance Use Risk Profile Scale showing incremental validity over the NEO Personality Inventory. Attentional biases to emotional faces did not characterise or predict mental disorders examined in the current sample.

**Discussion:**

Personality traits can indicate concurrent and prospective risk for mental disorders in a community youth sample, and identify at-risk youth beyond the impact of baseline symptoms. This study does not support the hypothesis that attentional biases mediate the relationship between personality and psychopathology in a community sample. Task and sample characteristics that contribute to differing results among studies are discussed.

## Introduction

Personality factors are consistently associated with psychopathology [1, 2] and measures of temperament in early childhood such as inhibition and impulsivity are found to predict psychopathology in adulthood [3, 4]. The nature of this relationship is, however, unclear. Cognitive vulnerability theories [5] posit that biases in attention (as well as memory and interpretation) contribute to the cause and maintenance of psychopathology. Personality is thought to impact the nature of an individual’s beliefs and attentional biases [6, 7]. For instance, anxious individuals can overestimate the likelihood of risk and danger and can be hypervigilant to indications of threat. They may adopt safety seeking behaviours such as avoidance as a response to their threat bias, which may in turn maintain the experience of anxiety or escalate into an anxiety disorder [6, 7]. A model of “personality neuroscience” [8] provides an opportunity to integrate trait views of personality with processing of cognitive or affective information, and suggests that information processing styles may mediate the relationship between personality and behaviour. Indeed, personality traits such as anxiety and aggressivity have been shown to influence neural responses to facial emotion processing in adults [9].

Understanding individuals’ sub-conscious processing styles and, in particular, biases in information processing, could help to elucidate their tendency to experience recurrent aversive emotional states. Attentional biases towards both positive and negative emotional cues are adaptive features of normal information processing that allow individuals to preferentially attend to emotionally-relevant stimuli. However, pronounced biases in emotional processing are also commonly seen across mental disorders such as depression, anxiety and externalising problems [10–12], with some suggesting that maladaptive functioning can be characterised as an exaggeration of normative [6, 13]. The evidence is, however, less well established within the developmental literature [12].

The emotional dot probe task is one of the most commonly used paradigms for assessing attentional biases to emotional stimuli [14], and involves the presentation of two words or pictures on a computer screen for a short interval of time, following which participants indicate the location of a dot. The task measures the time taken to respond to the appearance to the dot in the place of emotional (*i*.*e*. threatening) versus neutral stimuli. Difficulty in disengaging from threatening stimuli has been demonstrated in paediatric and adult anxious clinical and non-clinical populations through a meta-analysis of 172 studies [15]; in other words, anxious participants took longer to disengage from threatening stimuli than controls. Adults with depressive symptoms have been shown to demonstrate an attentional bias towards negative stimuli such as sad faces across 29 studies [16, 17]. With regards to the externalising spectrum, aggressive children and youth have been shown to pay more attention to aggressive than cooperative interactions [18] and impulsivity in 13–17 year-old youth has been associated with greater attentional biases towards angry relative to happy faces [10].

Whilst many studies support the commonly cited finding that anxious individuals have an attentional bias towards threatening stimuli [15], some do not support this association, which suggests that there are several important factors that may moderate this relationship. Firstly, findings in the childhood literature are less consistent than in studies on adults [14], suggesting that findings from the adult literature cannot necessarily be generalised to children. One reason for this may be limited attentional capacities, particularly in young children [19]. The task used to measure attentional bias is equally important. In the case of the dot probe task, some relevant factors are stimulus content and timing. Studies that uses words as target stimuli have reported a threat bias in anxious youth [20–23], whereas several studies that use picture stimuli such as faces did not [24, 25]. Conversely, a meta-analysis reported that results did not differ based on the use of word versus picture stimuli for the detection of a threat bias in anxious individuals [15]. The duration of stimulus exposure also impacts on the results obtained, as an attentional bias to threat is consistently detected at 500ms exposure [26], whereas attentional biases to depression are detected at longer exposures such as 1000ms [16]. The duration of stimulus exposure in the dot probe task associated with externalising problems or anger/hostility biases is, as yet, unclear [27]. The conditions during testing, and the mood of participants can also impact results, as some studies have noted that high state anxiety must be present in order to detect a threat bias in the dot-probe task in high trait-anxious children [28, 29]. Many studies also pre-select participants with high levels of the characteristics of interest (*e*.*g*. anxiety, depression, aggressivity), either in clinical or non-clinical ranges [15].

The role of attentional biases in healthy, community samples of youth has been examined less often than in clinical samples. Community samples allow us to evaluate the potential role of attentional biases in indicating prospective risk for the development of mental disorders, which could indicate a potential avenue for intervention for the onset of significant problems. Several authors have suggested that attentional biases, and the resulting impact on cognition, may partially mediate the relationship between temperament and mental disorders [30, 31]. The current study uses two personality measures to investigate this hypothesis in a youth community sample. The revised NEO Personality Inventory [32] assesses broad dimensions of personality, namely neuroticism, agreeableness, extraversion, openness and conscientiousness. Whilst they are not constructed as measures of psychopathology, some NEO traits are associated with mental health symptoms. Neuroticism, or the tendency to experience negative emotions, is considered by many to reflect the core of internalising problems, as well to be most important factor in behavioural public health, with its economic costs exceeding those associated with other psychiatric disorders [33]. A meta-analysis on the association between NEO personality traits and specific depressive, anxiety and substance use disorders revealed that all diagnostic groups were high on neuroticism and low on conscientiousness, and many disorders showed low extraversion [34]. Openness and agreeableness was largely unrelated to the analysed diagnoses. The Substance Use Risk Profile Scale (SURPS), on the other hand, was designed to and validated in the measurement of personality risk factors for the development of substance use and non-addictive psychopathology in youth and adults [35–37]. Namely, the internalising traits of hopelessness and anxiety sensitivity are risk factors for depressive and anxiety disorders, respectively [37, 38]. High levels of impulsivity, on the other hand, are associated with disinhibition over a range of behaviours, including antisocial tendencies [39] and Attention-Deficit/Hyperactivity Disorder [40]. Lastly, sensation seeking is associated with risk-taking behaviours for enhancement purposes, rather than with any particular form of psychopathology. A recent article has shown that using these four personality subscales on the SURPS can identify a high number of those who will develop substance use or mental health problems over 18 months, with sensitivity scores ranging from 72–91% [35]. Each scale is differentially related to specific mental health problems, and good specificity was obtained when examining the association between each personality scale and problematic outcomes. 70–80% of 14-year olds identified as high-risk by the impulsivity scale developed conduct or drug use problems within the next 18 months, and odds of developing severe depressive symptoms (for individuals prone to hopelessness) or emotional problems (for youth with high levels of anxiety sensitivity) over 18-months were 3–3.5 times greater than in youth without these high-risk profiles. One important practical advantage of the SURPS relative to the NEO is the length of the measure (23 versus 240 items), and therefore the ease of completion and potential for use as a systematic screening tool.

The current paper will present data from the IMAGEN study [41], the first multicentre study allowing an in-depth evaluation of risk phenotypes for mental disorders in adolescence using neuropsychological, self- and parent-report data on adolescents (as well as genetic and neuroimaging data, which will not be presented here). This detailed assessment in a large sample (N = 2257) distinguishes this sample from many others, and the longitudinal design allows an examination of prospective risk factors in a community sample. Specifically, this paper will explore whether attentional biases to emotional faces can identify youth at risk for mental disorders, and whether attentional biases to emotional faces overlap with known personality risk factors for psychopathology, and mediate the relationship between personality and psychopathology. The study also provides the opportunity to examine incremental validity of a measure of personality risk for psychopathology (the SURPS) relative to measures of normal variation in personality (the revised NEO).

## Materials and Methods

### Participants and procedure

Data from this project were obtained as part of the IMAGEN study, a multi-national research project coordinated by the Institute of Psychiatry, King’s College London. IMAGEN was conducted in 8 European sites across the United Kingdom, Ireland, France and Germany. 2257 14-year old adolescents and their parents were recruited from schools and using an internet recruitment site (www.imagen-info.com). Geographical areas were selected to maximise ethnic homogeneity, due to the genetic component of the IMAGEN study (not reported here). However, both private and state-funded schools were targeted in order to obtain a diverse sample of socioeconomic status, emotional and cognitive development. Written parental and child consent were required from all participants, following which adolescents were invited to complete a home-based assessment, and both adolescents and parents were invited to come to the local research centre for testing. The recruitment procedure and study protocol was approved by the KCL (King’s College London) College Research Ethics Committee CREC/06/07-71. The data presented focus on three tasks from a larger assessment battery. 1602 participants were re-assessed at 16 years of age through a home-based assessment.

### Measures

Psytools software (Delosis Ltd, London, UK) was used to assess participants’ personality, symptoms of mental disorders and emotional biases via its internet-based platform. Whilst personality and emotional biases were measured as part of a home assessment package, symptoms of mental disorders were assessed at the research facilities.

### Personality

#### Revised NEO Personality Inventory (NEO-PI-R)

The NEO-PI-R [32] is a 240-item questionnaire that assesses the “Big Five” dimensions of personality, namely Neuroticism, Extraversion, Openness, Agreeableness and Conscientiousness [42]. The NEOPI-R is validated as a method of assessing broad dimensions of personality in adolescence [43, 44]. Whilst it is not a measure of psychopathology, some NEO traits are related to mental health symptoms. A meta-analysis on the association between NEO personality traits and specific depressive, anxiety and substance use disorders revealed that all diagnostic groups were high on neuroticism and low on conscientiousness, and many disorders were associated with low extraversion [34]. Openness and agreeableness were largely unrelated to the analysed diagnoses.

#### Substance Use Risk Profile Scale (SURPS)

The SURPS [37] is a 23-item questionnaire assessing variation in personality risk for substance abuse and non-substance related pathology along 4 dimensions: sensation seeking, impulsivity, anxiety sensitivity and hopelessness. This scale has good concurrent, predictive and incremental validity (relative to other personality measures) with regards to differentiating individuals prone to reinforcement-specific patterns of substance-use, and is concurrently and prospectively associated with substance misuse and non-substance-related externalising behaviours and internalising symptoms [35–37]. The scale was translated, piloted and validated at the French and German sites before use.

### Development and Well-Being Assessment (DAWBA)

The DAWBA is a computer-based package of questionnaires, interviews, and rating techniques designed to generate DSM-IV-TR (and ICD-10) psychiatric diagnoses for 5–16-year-olds. The DAWBA has been validated in community and clinical samples, and diagnoses were consistent with those reported in patients’ case files [45]. The DAWBA has been validated in German [46], and the French version was developed for IMAGEN in cooperation with the author Robert Goodman. Both the child and parent were asked to respond, separately, to questions regarding the child’s psychiatric symptoms, under the supervision of a research assistant. These different types of information were brought together by a computer program which predicted the likelihood of diagnosis from 0 (<0.1%) to 5 (>70%). These likelihood scores were used as an indication of symptom severity. Due to the low prevalence rates of each of these disorders in this community sample, only those disorders which had at least 3% of the sample reporting at least a 15% likelihood of diagnosis at baseline were used for these analyses, namely Generalised Anxiety Disorder (GAD; 6.6%), Major Depressive Disorder (MDD; 3.7%), Attention-Deficit/Hyperactivity Disorder (ADHD; 6.0%), Oppositional Defiant Disorder (ODD; 5.8%) and Conduct Disorder (CD; 8.2%). DAWBA scores were reassessed 2 years post-baseline using a web-based assessment, completed by both children and parents from home.

### Emotional dot-probe task

This task is a variant of MacLeod, Mathews & Tata’s [47] widely used dot-probe task, and assesses attentional bias for emotional stimuli. Happy, angry and fearful facial expressions were used as emotional variants. 20 adult, greyscale IDs were selected from the MacBrain NimStim Face Stimulus Set (12 European-America, 8 African-American), and neutral faces of the same ID were used as matching stimuli. Participants were asked to respond indicating which side the probe was on using a computer keyboard. Trials were congruent (probe appeared behind the emotional face) or incongruent (probe appearing behind the neutral face). Each ID appeared once in each emotion in both types of trial (20x3x2 = 120 trials). The probe position (left vs right) was counter-balanced over the whole task and within each emotion condition. Although there is some debate as to whether words or picture stimuli are more sensitive measures of attentional biases, a meta-analysis of 172 studies reported that threat biases in anxious individual were equally consistent when measured with words or picture stimuli [15]. Face stimuli were displayed for 1000ms, following which the screen was cleared and participants’ reaction times were measured. The emotional faces were presented head-on, although given that this data was completed as part of a home assessment, it was not possible to control for the participants’ positioning relative to the computer during the task. Three attentional bias scores (anger, fear, happiness) were computed by subtracting the mean reaction times to congruent from incongruent probes for each emotion, with a positive bias indicating a greater reaction time latency to the emotional face. A fourth, general attentional bias score was calculated by obtaining the mean attentional bias across the three other emotions- in other words, this variable reflected the level of bias towards emotional stimuli, irrespective of valence (angry, fearful or happy). This study chose a 1000ms stimulus exposure in order to be more sensitive to depression-related [16], as less is known about this topic than threat-related biases in anxiety, which have more often been reported and are more reliably detected at 500ms exposure [26]. The optimal stimulus exposure to detect biases towards emotional faces associated with anger/hostility or externalising problems is, as yet, unknown [27], thus this did not impact the selection of the stimulus exposure.

### Data validation and exclusion criteria

A number of validation checks were completed during the home assessment, *i*.*e*. asking participants whether they were in a hurry, distracted or being watched by others. If participants responded positively to these validation checks, or if their responses were doubtful (*e*.*g*. with mean reaction times <100ms or if all responses were the same), their data was considered unreliable. These stringent reliability procedures were considered necessary for data completed as part of the home assessment as the research team sought to ensure that all data analysed was collected under similar conditions. Based on these validation checks, data for 191 participants (8.5%) were flagged as being unreliable based on their response to the SURPS, and 54 (2.39%) were flagged as being unreliable based on their responses to the dot probe task according to these criteria. In addition, participants whose mean reaction times were shorter than 200ms or longer than 2000ms on the dot probe task were also removed from the data, following recommendations by Koster, Crombez, Verschuere & De Houser [48]- this was the case for 69 participants (3.0%). The adjusted sample size was 1997 youth at baseline, and 1497 youth at follow-up. Being flagged for unreliable data at baseline was not predicted by any individual-level variables (*e*.*g*. gender, personality, attentional biases or mental health symptoms), however unreliable data differed significantly across recruitment sites (p<.001), with French sites having the smallest percentage of unreliable data (8.8%), followed by German sites (9.2%), then English-speaking sites (10.1%).

### Data analysis

Data were analysed using IBM SPSS Statistics version 21 (IBM corp, Armonk, NY) and STATA SE 13.1 (StataCorp, College Station, TX). The associations between personality trait levels, attentional biases and the likelihood of diagnosis of mental disorders at 14 years were examined using Pearson’s correlation analyses with Bonferroni adjustments for multiple comparisons. Separate hierarchical linear regressions were conducted in STATA to examine the prospective relationship between personality trait levels and attentional biases at 14 years with the risk for mental health symptoms at 16 years (*i*.*e*. GAD, MDD, ADHD, ODD, CD). Regression analyses accounted for recruitment sites as a cluster variable, and non-independence observations were adjusted for using tests based on the Huber-White sandwich estimate of variance [49]. This method provides standard errors that are robust for within-cluster (within-site) correlation. Gender and baseline mental health symptoms were used as covariates in the analyses. Linear regressions were then repeated without including NEO personality factors, in order to examine the incremental validity of the SURPS measure. Lastly, regression analyses were repeated without NEO or SURPS personality variables in order to investigate the capacity of attentional biases towards emotional faces to predict symptom severity without accounting for personality.

## Results

### Attrition

Attrition at follow-up was not predicted by gender, mental health symptoms or emotional biases. However, rates of follow-up differed significantly across recruitment sites (p<.001), with the highest follow-up rates in the English-speaking sites (81.2%), followed by the German sites (70%), then the French sites (51.9%). Participants with higher levels of impulsivity (p = .01), higher levels of sensation-seeking (p = .02), lower levels of openness (p = .003) and a higher likelihood of CD diagnosis (p = .01) were less likely to complete the follow up assessment. Other personality variables and mental health symptoms did not impact on the likelihood of participant attrition. Regression analyses were performed accounting for effects of gender, baseline mental health symptoms, personality and attentional biases. In order to account for baseline differences between recruitment sites, regression analyses in STATA accounted for recruitment sites as a cluster variable.

Please see Table 1 for reaction time data for the dot probe task according to emotional valence and trial type.

**Table 1:** Dot probe task reaction times by emotional stimulus and trial type at 14 years.

Emotional face	Trial type*	Reaction time in milliseconds
		Mean (standard deviation)
Anger	Congruent	463.90 (109.20)
	Incongruent	466.29 (114.84)
Fear	Congruent	467.00 (110.90)
	Incongruent	465.35 (113.09)
Happiness	Congruent	465.83 (112.57)
	Incongruent	464.92 (114.91)
General	Congruent	465.19 (100.74)
	Incongruent	464.84 (103.36)

*In congruent trials, the probe appeared behind the emotional face. In incongruent trials the probe appearing behind the neutral face.

### Association between personality, attentional bias to emotional faces and symptoms of mental disorders

Correlation analyses were conducted using Bonferroni-adjusted alpha levels of .0004 per test (.05/121). Impulsivity and hopelessness were positively correlated with risk for all mental health diagnoses at 14 years, and agreeableness and conscientiousness were negatively correlated with the risk for diagnoses (*p*<.004). Anxiety-sensitivity was associated with risk for internalising disorders (GAD and MDD), and neuroticism were associated with risk for all disorders except CD (*p*<.004). Sensation-seeking and extraversion were negatively associated with the risk of GAD diagnosis, and extraversion was also negatively correlated with the risk for MDD diagnosis (*p*<.004). Openness was negatively associated with the risk for CD diagnosis at 14 years. Neither personality traits nor mental health symptoms at 14 years were correlated with attentional biases. Please see Table 2 for further details. The current data did not fulfill the necessary conditions to test whether emotion processing mediate the effects of personality on mental disorders [50] as, although the independent variable (in this case, personality) was related to the dependent variable (psychopathology), the mediator (attentional bias) was related to neither the dependent nor independent variables. Therefore, mediation analyses were not conducted.

**Table 2:** Pearson’s *r* correlation values between emotional biases, personality traits and mental health symptoms at 14 years.

		Emotional bias				SURPS traits				NEO-FFI traits				
		Anger	Fear	Happy	General	H	AS	IMP	SS	Neur	Ext	Open	Agree	Cons
**DAWBA**	**GAD**	-.02	-.01	-.02	-.03	.24*	.19*	.11**	-.08*	.39*	-.17*	.05	-.14*	-.07*
	**MDD**	.03	-.04	.004	-.01	.25*	.08*	.18**	.004	.33*	-.11*	.03	-.20*	-.08*
	**ADHD**	.02	.05	.02	.04	.14*	-.03	.23*	.05	.08*	-.003	-.04	-.19*	-.26*
	**ODD**	.01	.04	.04	.05	.11*	-.02	.17*	.01	.10*	-.07	-.06	-.18*	-.17*
	**CD**	.01	.01	.04	.03	.10*	.002	.23*	.05	.06	.03	-.08*	-.26*	-.20*
**SURPS**	**H**	.02	.01	.01	.01	-	-	-	-	.49*	-.38*	-.07*	-.29*	-.38*
	**AS**	-.02	.02	-.03	-.02	-	-	-	-	.35*	-.07	.07*	-.01	.04
	**IMP**	.02	.02	.01	.02	-	-	-	-	.28*	.11*	-.11*	.43*	-.35*
	**SS**	-.002	.01	-.02	-.01	-	-	-	-	-.09*	.19*	.17*	-.06	-.04
**Emotional bias**	**Anger**	-	-	-	-	-	-	-	-	.01	-.03	-.01	-.02	-.04
	**Fear**	-	-	-	-	-	-	-	-	-.02	-.003	.01	-.03	-.04
	**Happy**	-	-	-	-	-	-	-	-	-.02	-.01	.004	-.02	-.02
	**General**	-	-	-	-	-	-	-	-	-.02	-.02	.01	-.04	-.05

Note. *p<.0004 (significance level adjusted for multiple comparisons using the Bonferroni test)

DAWBA = Development and Well-Being Assessment, GAD = Generalised Anxiety Disorder; MDD = Major Depressive Disorder; ADHD = Attention-Deficit/Hyperactivity Disorder, ODD = Oppositional Defiant Disorder; CD = Conduct Disorder, SURPS = Substance Use and Risk Profile Scale, H = Hopelessness, AS = Anxiety-Sensitivity; IMP = Impulsivity, SS = Sensation-Seeking, NEO-FFI = NEO Five-Factor Inventory; Neur = Neuroticism, Extr = Extraversion, Open = Openness, Agree = Agreeableness, Cons = Conscientiousness

### Predictors of mental disorders at 16 years

Hopelessness (*p* = .002), neuroticism (*p*<.001), openness (p = .003) and conscientiousness (*p* = .007) positively predicted the likelihood of GAD diagnosis at 16 years, over and above baseline symptom levels. Neuroticism predicted an increased likelihood of MDD diagnosis at 16 years (*p* = .04). Extraversion predicted an increased likelihood of ADHD diagnosis (*p* = .03). SURPS traits did not predict MDD or ADHD when controlling for baseline symptom levels and NEO traits. Impulsivity predicted an increased likelihood of ODD diagnosis at 16 years (*p* = .04), and agreeableness at 14 years old was negatively associated with ODD at 16 years (*p* = .01). Impulsivity (*p* = .02) and hopelessness (*p* = .02) at 14 years predicted an increased likelihood of CD diagnosis at 16 years, but NEO traits did not. When NEO personality factors were removed from the regression model, impulsivity at 14 years predicted MDD diagnosis at 16 years (β = .09, S.E. = .02, *p* = .002), and anxiety sensitivity at 14 years predicted GAD diagnosis at 16 years (β = .08, S.E. = .01, *p* <.001). No other results changed. Attentional biases towards emotional faces at 14 years did not predict mental disorder diagnosis at 16 years for any of the symptoms examined. Results remained unchanged when personality variables were removed from the regression models. Please see Table 3 for details.

**Table 3:** Hierarchical linear regressions predicting symptom severity at 16 years, accounting for site as cluster.

Baseline covariates (14 years)	Symptom severity at 16 years. β (SE)				
	GAD	MDD	ADHD	ODD	CD
Gender	.47(.02)***	.38(.04)***	-.20(.06)*	.01(.07)	-.03(.06)
GAD	.34(.05)***	.12(.03)**	-.03(.02)	.001(.03)	-.02(.03)
MDD	.12(.04)	.23(.03)***	.08(.04)	.04(.03)	.06(.02)*
ADHD	.07(.02)*	.07(.04)	.52(.04)***	.14(.04)**	.08(.03)*
ODD	.003(.04)	-.03(.04)	.04(.04)	.29(.04)***	.09(.02)**
CD	-.02(.03)	.07(.04)	.09(.05)	.12(.03)**	.34(.04)***
H	.10(.02)**	.06(.06)	.05(.04)	.02(.03)	.11(.04)*
AS	.04(.01)	-.02(.02)	.003(.03)	-.05(.02)	-.01(.02)
IMP	.04(.04)	.05(.03)	.004(.03)	.05(.02)*	.09(.03)*
SS	-.02(.02)	-.01(.02)	-.03(.04)	-.02(.03)	-.003(.04)
Neur	.14(.02)***	.08(.03)*	-.01(.02)	.03(.03)	-.01(.03)
Extr	.03(.02)	.004(.02)	.07(.02)*	.01(.02)	.06(.03)
Open	.08(.02)**	.05(.03)	.01(.03)	.03(.02)	.03(.03)
Agree	-.04(.02)	-.07(.03)	-.03(.03)	-.11(.03)**	-.05(.03)
Cons	.10(.03)**	-.02(.03)	-.05(.03)	.04(.03)	.02(.02)
Anger bias†	-.06(.04)	-.02(.05)	-.03(.05)	.04(.03)	-.06(.06)
Fear bias†	.02(.05)	-.10(.06)	.12(.06)	.04(.06)	.02(.06)
Happy bias†	.04(.08)	-.11(.06)	-.08(.09)	.03(.13)	.002(.08)
General emotional bias†	0	0	0	0	0

Note. β = Standardised beta; SE = Standard Error

***p≤.001

**p ≤ .01

*p < .05

GAD = Generalised Anxiety Disorder; MDD = Major Depressive Disorder; ADHD = Attention-Deficit/Hyperactivity Disorder, ODD = Oppositional Defiant Disorder; CD = Conduct Disorder, H = Hopelessness, AS = Anxiety-Sensitivity; IMP = Impulsivity, SS = Sensation-Seeking, Neur = Neuroticism, Extr = Extraversion, Open = Openness, Agree = Agreeableness, Cons = Conscientiousness

† Results remained unchanged when personality was removed from the model.

## Discussion

The aim of this paper was to investigate the role of personality factors and attentional biases towards emotional faces in the risk for mental disorder diagnosis in adolescence, both concurrently and over two years, and to evaluate whether attentional biases towards emotional faces can be used to identify youth at risk for mental disorders in a community youth sample that has not been pre-selected for high levels of traits or symptoms of interest (*e*.*g*. anxiety, depression, externalising problems). Attentional biases towards emotional faces at 14 years did not concurrently or prospectively predict the likelihood of being diagnosed with GAD, MDD, ADHD or CD, suggesting that attentional biases as measured by the dot probe task in the current study do not reliably identify early risk for developing mental disorders in community youth. These results held true when considering attentional biases towards particular emotional faces (angry, happy, fearful) as well as a general attentional bias towards emotions, irrespective of valence. Attentional biases also did not mediate the relationship between personality factors and mental health symptoms. This suggests that, in a healthy community sample, early symptoms of mental disorders are more reliably detected using personality measures than attentional biases to emotional faces. Dysregulation in attentional biases towards emotions may, instead, be present in individuals with a current psychiatric disorder or adults with high levels of certain symptoms (*e*.*g*. anxiety; [15]).

These null findings contrast with some previous research [15, 17], but are supported by others [24, 25]. Stimulus latency is known to impact on the detection of attentional biases, and our stimulus exposure duration of 1000ms may have been too long to detect an attentional bias to threat (which was expected in youth high in anxiety sensitivity, for instance)- threat biases are more consistently detected at 500ms [26]. However, a 1000ms duration was optimised in order to detect of potential attentional biases in depression-prone youth, following Gotlib and colleagues [16]. Thus, the fact that attentional biases were not associated with personality traits related to depression (*e*.*g*. hopelessness, neuroticism), or the likelihood of MDD diagnosis suggests that attentional biases cannot be used to indicate risk for depression. The optimal duration of stimulus exposure to identify attentional biases associated with impulsivity or externalising problems using the dot-probe task has not been established thus far, to the authors’ knowledge. The results of this study suggest that attentional biases measured using a 1000ms exposure cannot be used to identify risk factors for externalising problems. One possible explanation for the null findings could be that our outcome measure of mental disorder symptoms was not sensitive enough. However, the fact that personality measures were predictive of outcome suggests that the DAWBA was sufficiently sensitive and specific. The findings from this study supported the concurrent and prospective predictive validity of personality traits in indicating risk for mental disorders. There was some cross-sectional specificity between personality traits and mental health symptoms at 14 years (*i*.*e*. extraversion was negatively associated with GAD and MDD, but not associated with ADHD, ODD or CD), but hopelessness, impulsivity, neuroticism, low agreeableness and low conscientiousness emerged as relatively general indicators of risk for externalising and internalising problems. However, greater specificity emerged over time, with neuroticism at 14 years being the only personality trait to predict an increased likelihood of MDD diagnosis at 16 years, and impulsivity and low agreeableness at 14 years predicting ODD diagnosis at 16 years. These results are fairly consistent with other studies [34, 35]. SURPS traits showed incremental validity over the NEO in predicting the likelihood of GAD, ODD and CD diagnosis at 16 years, but not MDD or ADHD diagnoses. This highlights the value of the SURPS as a short and easy-to-administrate measure in predicting the risk for mental disorders over and above more cumbersome questionnaires such as the NEO [37].

There are several limitations of the current study. Firstly, due to the 1000ms duration of stimulus presentation during the dot probe task, it is not possible to conclude whether the absence of threat-related biases is a feature of the sample or the measure. It would thus be recommended to repeat the dot probe task in a community sample with a 500ms exposure duration. Similarly, it would be informative to measure attentional biases towards anger and hostility using a 500ms exposure to assess whether a shorter stimulus exposure could reveal associations with risk for externalising problems. However, the absence depression-related biases can be attributed to the sample, as other studies have detected biases at a 1000ms duration. Future studies should consider using a range of timings (*e*.*g*. 500ms, 1000ms, 1500ms) to disentangle questions of the appropriate time frame to measure attentional biases towards particular emotions. For instance, some studies have noted that a longer response time results in avoidance of threatening stimuli in anxiety-prone individuals, whereas a bias towards threat is typically found at shorter (*e*.*g*. 500ms) stimuli durations [48, 51]. A recent study has suggested that emotional attention is context and time dependent, and assessing the time-varying nature of attentional biases can predict symptoms of mental disorders more reliably than the traditional bias score as currently measured [52]. In a related comment, some studies have noted that attentional biases are more likely to be expressed in situations where individuals are emotionally aroused (*e*.*g*. stressed), suggesting that attentional biases influence mood through their interaction with the environment [53]. Indeed, it is increasingly acknowledged that cognitive processes such as attention, memory and interpretation are related to one another and to emotion regulation [54], and that attentional biases should be considered in relation to other cognitive processes [55]. Whilst this was beyond the scope of the current study, an investigation of the interactions between attentional biases, memory, interpretation and mood, as well as the time-varying nature of attentional biases in relation to personality and psychopathology would be worthy of future study. A second limitation is that the dot-probe task used here employs adult grey scale faces and thus may not adequately reflect the social context of youth. Thirdly, using DAWBA computer-rated likelihood scores as a proxy for symptom severity is not equivalent to having symptoms rated by clinicians, however this computer-rated approach has been validated as an adequate manner to evaluate symptom severity [46]. A comparison of clinician and computer ratings in a subsample of 343 youth also showed that ratings were significant correlated (*p*<.001) with Pearson’s *r*s in the moderate range [0.35 (GAD)- 0.6 (ODD)]. Fourth, there were differences between recruitment sites in terms of attrition and the likelihood of being flagged for unreliable data. This likely reflects site-specific protocols, and these differences were taken into account in the analyses by conducting cluster based regressions accounting for site. Lastly, the correlations between DAWBA diagnoses and SURPS scores, though significant, are small. The fact that the sample in question was healthy is a strength and a limitation of the study in that there was relatively little mental illness to predict. However, the sample characteristics allowed us to investigate the role of personality and attentional biases towards emotional faces on the risk of mental health diagnosis in a community sample. Other strengths of this study include the detailed data collection across multiple modalities (*e*.*g*. here, self-report, parent-report and computer tasks) with a large sample across multiple European sites, comprehensive assessment of personality using two well-validated measures, computer and clinician-validated assessment of mental disorder symptoms from both the child and parent’s perspectives, as well as the longitudinal design providing an opportunity to examine prospective risk factors for mental disorders.

In conclusion, these findings suggest that the traditional measure of attentional biases towards emotional faces using the dot-probe task does not reliably predict the risk for developing mental health problems in a community sample. Instead, this study highlights instead both “big five” and SURPS personality factors that differentially indicate prospective risk for mental disorders, and suggests that the SURPS has incremental validity over the NEO-PI-R in predicting risk for GAD, ODD and CD diagnoses. These findings suggest that, while attentional biases to emotional faces may characterise participants with high levels of anxiety, depression or hostility, they are not pre-cursors to the development of mental disorders. This suggests that the risk for mental health problems in a community sample may be characterised, not by attentional biases, but in other ways, such as behavioural, cognitive or motivational tendencies. It is well established, for instance, that personality traits predict coping strategies [56], and it may be that specific cognitive or behavioural tendencies under stressful conditions mediate personality vulnerability to psychiatric disorders. The current study suggests that personality factors appear more implicated in risk for the development of psychiatric disorders, possibly through other cognitive-behavioural indicators such as reinforcement learning or response inhibition [57, 58]. This suggests that preventive interventions should target personality risk factors or coping styles, and indeed some selective approaches have demonstrated that interventions focused on SURPS personality risk factors do indeed decrease the risk for future substance use problems or mental disorders [59, 60].

## References

[1] Krueger RF, Tackett JL. Personality and psychopathology: working toward the bigger picture. J Pers Disord. 2003;17(2):109–28. 12755325 10.1521/pedi.17.2.109.23986

[2] Maher BA, Maher WB. Personality and psychopathology: a historical perspective. J Abnorm Psychol. 1994;103(1):72–7. 8040484 10.1037//0021-843x.103.1.72

[3] Caspi A, Moffitt TE, Newman DL, Silva PA. Behavioral observations at age 3 years predict adult psychiatric disorders. Longitudinal evidence from a birth cohort. Arch Gen Psychiatry. 1996;53(11):1033–9. 8911226 10.1001/archpsyc.1996.01830110071009

[4] Kagan J, Snidman N. Infant predictors of inhibited and uninhibited profiles. Psychol Sci 1991;2(1):40–4.

[5] Beck AT. Cognitive models of depression. J Cogn Psychother Int Q. 1987;1:5–37.

[6] Beck AT, Haigh EA. Advances in cognitive theory and therapy: the generic cognitive model. Annu Rev Clin Psychol. 2014;10:1–24. 10.1146/annurev-clinpsy-032813-153734 24387236

[7] Clark DA, Beck AT, Alford BA. Scientific foundations of cognitive therapy and therapy of depression New York: John Wiley & Sons; 1999.

[8] Canli T. Functional brain mapping of extraversion and neuroticism: learning from individual differences in emotion processing. J Pers. 2004;72(6):1105–32. 15509278 10.1111/j.1467-6494.2004.00292.x

[9] Calder AJ, Ewbank M, Passamonti L. Personality influences the neural responses to viewing facial expressions of emotion. Philos Trans R Soc Lond B Biol Sci. 2011;366(1571):1684–701. 10.1098/rstb.2010.0362 21536554 PMC3130379

[10] D'Acremont M, Van Der Linden M. Memory for angry faces, impulsivity, and problematic behavior in adolescence. J Abnorm Child Psych. 2007;35(2):313–24. 17243017 10.1007/s10802-006-9092-1

[11] Mathews A, MacLeod C. Cognitive vulnerability to emotional disorders. Annu Rev Clin Psychol. 2005;1:167–95. 17716086 10.1146/annurev.clinpsy.1.102803.143916

[12] Waters AM, Neumann DL, Henry J, Craske MG, Ornitz EM. Baseline and affective startle modulation by angry and neutral faces in 4-8-year-old anxious and non-anxious children. Biol Psychol. 2008;78(1):10–9. 10.1016/j.biopsycho.2007.12.005 18243481

[13] Yiend J. The effects of emotion on attention: A review of attentional processing of emotional information. Cognition Emotion. 2010;24(1):3–47.

[14] Puliafico AC, Kendall PC. Threat-related attentional bias in anxious youth: a review. Clin Child Fam Psychol Rev. 2006;9(3–4):162–80. 17053961 10.1007/s10567-006-0009-x

[15] Bar-Haim Y, Lamy D, Pergamin L, Bakermans-Kranenburg MJ, Van Ijzendoorn MH. Threat-related attentional bias in anxious and nonanxious individuals: A meta-analytic study. Psychol Bull. 2007;133(1):1–24. 17201568 10.1037/0033-2909.133.1.1

[16] Gotlib IH, Krasnoperova E, Yue DN, Joormann J. Attentional biases for negative interpersonal stimuli in clinical depression. J Abnorm Psychol. 2004;113(1):121–35. 14992665 10.1037/0021-843X.113.1.121

[17] Peckham AD, McHugh RK, Otto MW. A meta-analysis of the magnitude of biased attention in depression. Depress Anxiety. 2010;27(12):1135–42. 10.1002/da.20755 21049527

[18] Schultz D, Izard CE, Bear G. Children's emotion processing: relations to emotionality and aggression. Dev Psychopathol. 2004;16(2):371–87. 15487601 10.1017/s0954579404044566

[19] Brodeur DA, Boden C. The effects of spatial uncertainty and cue predictability on visual orienting in children. Cognitive Dev. 2000;15(3):367–82.

[20] Dalgleish T, Moradi AR, Taghavi MR, Neshat-Doost HT, Yule W. An experimental investigation of hypervigilance for threat in children and adolescents with post-traumatic stress disorder. Psychol Med. 2001;31(3):541–7. 11305862 10.1017/s0033291701003567

[21] Dalgleish T, Taghavi R, Neshat-Doost H, Moradi A, Canterbury R, Yule W. Patterns of processing bias for emotional information across clinical disorders: a comparison of attention, memory, and prospective cognition in children and adolescents with depression, generalized anxiety, and posttraumatic stress disorder. J Clin Child Adolesc Psychol. 2003;32(1):10–21. 12573928 10.1207/S15374424JCCP3201_02

[22] Taghavi MR, Neshat-Doost HT, Moradi AR, Yule W, Dalgleish T. Biases in visual attention in children and adolescents with clinical anxiety and mixed anxiety-depression. J Abnorm Child Psychol. 1999;27(3):215–23. 10438187 10.1023/a:1021952407074

[23] Vasey MW, Daleiden EL, Williams LL, Brown LM. Biased attention in childhood anxiety disorders: a preliminary study. J Abnorm Child Psychol. 1995;23(2):267–79. 7642837 10.1007/BF01447092

[24] Pine DS, Klein RG, Mannuzza S, Moulton JL 3rd, Lissek S, Guardino M, et al Face-emotion processing in offspring at risk for panic disorder. J Am Acad Child Adolesc Psychiatry. 2005;44(7):664–72. 15968235 10.1097/01.chi.0000162580.92029.f4

[25] Waters AM, Lipp OV, Spence SH. Attentional bias toward fear-related stimuli: An investigation with nonselected children and adults children with anxiety disorders. J Exp Child Psychol. 2004;89(4 SPEC.ISS.):320–37. 15560877 10.1016/j.jecp.2004.06.003

[26] Frewen PA, Dozois DJ, Joanisse MF, Neufeld RW. Selective attention to threat versus reward: meta-analysis and neural-network modeling of the dot-probe task. Clin Psychol Rev. 2008;28(2):307–37. 17618023 10.1016/j.cpr.2007.05.006

[27] Faunce GJ, Mapledoram PK, Soames Job RF. Type A behaviour pattern and attentional bias in relation to anger/hostility, achievement, and failure. Pers Indiv Differ. 2004;36(8):1975–88.

[28] Heim-Dreger U, Kohlmann CW, Eschenbeck H, Burkhardt U. Attentional biases for threatening faces in children: vigilant and avoidant processes. Emotion. 2006;6(2):320–5. 16768563 10.1037/1528-3542.6.2.320

[29] Rutherford E, MacLeod C, Campbell L. Negative selectivity effects and emotional selectivity effects in anxiety: Differential attentional correlates of state and trait variables. Cognition Emotion. 2004;18(5):711–20.

[30] Canli T, Zhao Z, Desmond JE, Kang E, Gross J, Gabrieli JD. An fMRI study of personality influences on brain reactivity to emotional stimuli. Behav Neurosci. 2001;115(1):33–42. 11256451 10.1037/0735-7044.115.1.33

[31] Lonigan CJ, Vasey MW, Phillips BM, Hazen RA. Temperament, anxiety, and the processing of threat-relevant stimuli. J Clin Child Adolesc Psychol. 2004;33(1):8–20. 15028537 10.1207/S15374424JCCP3301_2

[32] Costa PT Jr, McCrae RR. Normal Personality Assessment in Clinical Practice: The NEO Personality Inventory. Psychol Assessment. 1992;4(1):5–13.

[33] Cuijpers P, Smit F, Penninx BW, de Graaf R, ten Have M, Beekman AT. Economic costs of neuroticism: a population-based study. Arch Gen Psychiatry. 2010;67(10):1086–93. 10.1001/archgenpsychiatry.2010.130 20921124

[34] Kotov R, Gamez W, Schmidt F, Watson D. Linking "big" personality traits to anxiety, depressive, and substance use disorders: a meta-analysis. Psychol Bull. 2010;136(5):768–821. 10.1037/a0020327 20804236

[35] Castellanos-Ryan N, O'Leary-Barrett M, Sully L, Conrod P. Sensitivity and specificity of a brief personality screening instrument in predicting future substance use, emotional, and behavioral problems: 18-month predictive validity of the substance use risk profile scale. Alcohol Clin Exp Res. 2013;37 Suppl 1:E281–90. 10.1111/j.1530-0277.2012.01931.x 22974180

[36] Krank M, Stewart SH, O'Connor R, Woicik PB, Wall AM, Conrod PJ. Structural, concurrent, and predictive validity of the Substance Use Risk Profile Scale in early adolescence. Addict Behav. 2011;36(1–2):37–46. 20826056 10.1016/j.addbeh.2010.08.010

[37] Woicik PA, Stewart SH, Pihl RO, Conrod PJ. The Substance Use Risk Profile Scale: a scale measuring traits linked to reinforcement-specific substance use profiles. Addict Behav. 2009;34(12):1042–55. 10.1016/j.addbeh.2009.07.001 19683400

[38] Stewart SH, Kushner MG. Introduction to the Special Issue on "Anxiety Sensitivity and Addictive Behaviors". Addict Behav. 2001;26(6):775–85. 11768544 10.1016/s0306-4603(01)00236-2

[39] Luengo MA, Carrillo-de-la-Pena MT, Otero JM, Romero E. A short-term longitudinal study of impulsivity and antisocial behavior. J Pers Soc Psychol. 1994;66(3):542–8. 8169763 10.1037//0022-3514.66.3.542

[40] Winstanley CA, Eagle DM, Robbins TW. Behavioral models of impulsivity in relation to ADHD: translation between clinical and preclinical studies. Clin Psychol Rev. 2006;26(4):379–95. 16504359 10.1016/j.cpr.2006.01.001 PMC1892795

[41] Schumann G, Loth E, Banaschewski T, Barbot A, Barker G, Buchel C, et al The IMAGEN study: reinforcement-related behaviour in normal brain function and psychopathology. Mol Psychiatry. 2010;15(12):1128–39. 10.1038/mp.2010.4 21102431

[42] McCrae RR, John OP. An introduction to the five-factor model and its applications. J Pers. 1992;60(2):175–215. 1635039 10.1111/j.1467-6494.1992.tb00970.x

[43] De Fruyt F, Mervielde I, Hoekstra HA, Rolland JP. Assessing adolescents' personality with the NEO PI-R. Assessment. 2000;7(4):329–45. 11172584 10.1177/107319110000700403

[44] McCrae RR, Costa PT Jr, Terracciano A, Parker WD, Mills CJ, De Fruyt F, et al Personality trait development from age 12 to age 18: Longitudinal, cross-sectional, and cross-cultural analyses. J Pers Soc Psychol. 2002;83(6):1456–68. 12500824

[45] Goodman R, Ford T, Richards H, Gatward R, Meltzer H. The Development and Well-Being Assessment: description and initial validation of an integrated assessment of child and adolescent psychopathology. J Child Psychol Psychiatry. 2000;41(5):645–55. 10946756

[46] Kuhn C, Winkler Metzke C, Aebi M, Steinhausen HC. PW01-59—Effects of an internet based assessment of child and adolescent psychopathology (DAWBA) on clinical decision making. Eur Psychiat. 2010;25, Supplement 1(0):1475.

[47] MacLeod C, Mathews A, Tata P. Attentional bias in emotional disorders. J Abnorm Psychol. 1986;95(1):15–20. 3700842 10.1037//0021-843x.95.1.15

[48] Koster EH, Crombez G, Verschuere B, De Houwer J. Selective attention to threat in the dot probe paradigm: differentiating vigilance and difficulty to disengage. Behav Res Ther. 2004;42(10):1183–92. 15350857 10.1016/j.brat.2003.08.001

[49] White H. A heteroskedasticity-consistent covariance matrix estimator and a direct test for heteroskedasticity. Econometrica 1980;48(817–838).

[50] Baron RM, Kenny DA. The moderator-mediator variable distinction in social psychological research: conceptual, strategic, and statistical considerations. J Pers Soc Psychol. 1986;51(6):1173–82. 3806354 10.1037//0022-3514.51.6.1173

[51] Mogg K, Bradley B, Miles F, Dixon R. Time course of attentional bias for threat scenes: Testing the vigilance‐avoidance hypothesis. Cognition Emotion. 2004;18(5):689–700.

[52] Zvielli A, Bernstein A, Koster EHW. Temporal dynamics of attentional bias. Clin Psychol Sci. 2014:1–17.

[53] MacLeod C, Rutherford E, Campbell L, Ebsworthy G, Holker L. Selective attention and emotional vulnerability: Assessing the causal basis of their association through the experimental manipulation of attentional bias. J Abnorm Psychol. 2002;111(1):107–23. 11866165

[54] Gotlib IH, Joormann J. Cognition and depression: current status and future directions. Annu Rev Clin Psychol. 2010;6:285–312. 10.1146/annurev.clinpsy.121208.131305 20192795 PMC2845726

[55] Everaert J, Duyck W, Koster EH. Attention, interpretation, and memory biases in subclinical depression: a proof-of-principle test of the combined cognitive biases hypothesis. Emotion. 2014;14(2):331–40. 10.1037/a0035250 24512247

[56] Connor-Smith JK, Flachsbart C. Relations between personality and coping: a meta-analysis. J Pers Soc Psychol. 2007;93(6):1080–107. 18072856 10.1037/0022-3514.93.6.1080

[57] Castellanos-Ryan N, Rubia K, Conrod PJ. Response inhibition and reward response bias mediate the predictive relationships between impulsivity and sensation seeking and common and unique variance in conduct disorder and substance misuse. Alcohol Clin Exp Res. 2011;35(1):140–55. 10.1111/j.1530-0277.2010.01331.x 21039636

[58] Mohr C, Braun S, Bridler R, Chmetz F, Delfino JP, Kluckner VJ, et al. Insufficient coping behavior under chronic stress and vulnerability to psychiatric disorders. Psychopathology. 2014. 10.1159/000356398 24435022

[59] Conrod PJ, O'Leary-Barrett M, Newton N, Topper L, Castellanos-Ryan N, Mackie C, et al Effectiveness of a selective, personality-targeted prevention program for adolescent alcohol use and misuse: a cluster randomized controlled trial. JAMA psychiatry. 2013;70(3):334–42. 10.1001/jamapsychiatry.2013.651 23344135

[60] O'Leary-Barrett M, Topper L, Al-Khudhairy N, Pihl RO, Castellanos-Ryan N, Mackie CJ, et al Two-year impact of personality-targeted, teacher-delivered interventions on youth internalizing and externalizing problems: a cluster-randomized trial. J Am Acad Child Adolesc Psychiatry. 2013;52(9):911–20. 10.1016/j.jaac.2013.05.020 23972693

